# Supplementary material for: Membrane lipid composition modulates the organization of VDAC1, a mitochondrial gatekeeper
Source: Commun Biol. 2025 Jun 17;8:936. doi: 10.1038/s42003-025-08311-5 (PMC12174345; doi:10.1038/s42003-025-08311-5)
Supplement: Supplementary file 2 — Description of Additional Supplementary Files [file 42003_2025_8311_MOESM2_ESM.docx]

Description of Additional Supplementary Files

**File name:** Supplementary Movie 1

**Description:** HS-AFM movie of a VDAC1 PC PE 2%Chol membrane absorbed on the mica substrate at 33ºC. Movie parameters: frame rate 969 ms; full image of 200 nm x 200 nm and 256x256 pixels; colour depth 8bit (256 values); full colour scale.

**File name:** Supplementary Data 1

**Description:** Source data for figure 5.
